# MUTUAL INFORMATION OF NEUROIMAGES AND GENETIC VARIANTS TO STUDY THE DETERMINANTS OF BRAIN AGING

**DOI:** 10.1093/geroni/igad104.3483

**Published:** 2023-12-21

**Authors:** Nicholas Kim, Anar Amgalan, Phoebe Imms, Nahian Chowdhury, Nikhil Chaudhari, Andrei Irimia

**Affiliations:** University of Southern California, Los Angeles, California, United States; University of Southern California, Los Angeles, California, United States; University of Southern California, Los Angeles, California, United States; University of Southern California, Los Angeles, California, United States; University of Southern California, Los Angeles, California, United States; University of Southern California, Los Angeles, California, United States

## Abstract

Understanding the genetic determinants of brain aging is important because advanced biological age of the brain is a risk factor for Alzheimer’s disease (AD). Single-nucleotide polymorphisms (SNPs) are genetic variations with effects on brain aging; mapping their influence on brain structure can elucidate the genetic correlates of AD. We adopted an information theoretic approach to this by computing the mutual information (MI) between (A) 22 AD-risk SNPs and (B) the MRI intensities of the cortex in 6,000 UK Biobank participants (~50% females) aged 54 to 84 years. These cortex-wide calculations quantified how SNPs impact brain structure. As expected, the APOE-ε4 allele, the strongest genetic risk factor for AD, exhibited significantly (p < 0.025) higher MI with MRI intensity, when compared to the average MI expected for a null distribution model, in the medial parietal lobe, pars orbitalis, and left inferior occipital lobe, three brain structures responsible for memory recall, language processing, and visual perception. These findings reflect the memory loss, decreased speech, and reduced peripheral vision experienced by individuals with AD. The ABCA7 allele, a risk factor for late- onset AD, had significantly (p < 0.025) higher MI in the inferior temporal sulcus and angular gyrus. These structures are responsible for visual recognition and attention, two functions that are severely impaired by AD. Other findings reveal similar relationships between AD-risk SNPs and brain structure. Our findings illustrate how mapping the MI between MRI intensities and SNPs provides insight into genetic influences on brain structures linked to AD symptoms.

